# Linear and Nonlinear Associations Between Vitamin D and Grip Strength: A Mendelian Randomization Study in UK Biobank

**DOI:** 10.1093/gerona/glac255

**Published:** 2022-12-25

**Authors:** Snehal M Pinto Pereira, Victoria Garfield, Thomas Norris, Stephen Burgess, Dylan M Williams, Richard Dodds, Avan A Sayer, Sian M Robinson, Rachel Cooper

**Affiliations:** Institute of Sport, Exercise and Health, Division of Surgery & Interventional Science, University College London, London, UK; MRC Unit for Lifelong Health and Ageing at UCL, London, UK; Institute of Sport, Exercise and Health, Division of Surgery & Interventional Science, University College London, London, UK; MRC Biostatistics Unit, University of Cambridge, Cambridge, UK; MRC Unit for Lifelong Health and Ageing at UCL, London, UK; AGE Research Group, Translational and Clinical Research Institute, Faculty of Medical Sciences, Newcastle University, Newcastle upon Tyne, UK; NIHR Newcastle Biomedical Research Centre, Newcastle University and Newcastle Upon Tyne Hospitals NHS Foundation Trust, Newcastle upon Tyne, UK; AGE Research Group, Translational and Clinical Research Institute, Faculty of Medical Sciences, Newcastle University, Newcastle upon Tyne, UK; NIHR Newcastle Biomedical Research Centre, Newcastle University and Newcastle Upon Tyne Hospitals NHS Foundation Trust, Newcastle upon Tyne, UK; AGE Research Group, Translational and Clinical Research Institute, Faculty of Medical Sciences, Newcastle University, Newcastle upon Tyne, UK; NIHR Newcastle Biomedical Research Centre, Newcastle University and Newcastle Upon Tyne Hospitals NHS Foundation Trust, Newcastle upon Tyne, UK; AGE Research Group, Translational and Clinical Research Institute, Faculty of Medical Sciences, Newcastle University, Newcastle upon Tyne, UK; NIHR Newcastle Biomedical Research Centre, Newcastle University and Newcastle Upon Tyne Hospitals NHS Foundation Trust, Newcastle upon Tyne, UK

**Keywords:** 25(OH)D, Grip strength, Mendelian randomization

## Abstract

**Background:**

Low vitamin D status is a widespread phenomenon. Similarly, muscle weakness, often indicated by low grip strength, is another public health concern; however, the vitamin D–grip strength relationship is equivocal. It is important to understand whether variation in vitamin D status causally influences muscle strength to elucidate whether supplementation may help prevent/treat muscle weakness.

**Methods:**

UK Biobank participants, aged 37–73 years, with valid data on Vitamin D status (circulating 25-hydroxyvitamin D [25(OH)D] concentration) and maximum grip strength were included (*N* = 368,890). We examined sex-specific cross-sectional associations between 25(OH)D and grip strength. Using Mendelian randomization (MR), we estimated the strength of the 25(OH)D–grip strength associations using genetic instruments for 25(OH)D as our exposure. Crucially, because potential effects of vitamin D supplementation on strength could vary by underlying 25(OH)D status, we allowed for nonlinear relationships between 25(OH)D and strength in all analyses.

**Results:**

Mean (*SD*) of 25(OH)D was 50 (21) nmol/L in males and females. In cross-sectional analyses, there was evidence of nonlinear associations between 25(OH)D and strength, for example, compared to males with 50 nmol/L circulating 25(OH)D, males with 75 nmol/L had 0.36 kg (0.31,0.40) stronger grip; males with 25 nmol/L had 1.01 kg (95% confidence interval [CI]: 0.93, 1.08) weaker grip. In MR analyses, linear and nonlinear models fitted the data similarly well, for example, 25 nmol/L higher circulating 25(OH)D in males was associated with 0.25 kg (−0.05, 0.55) greater grip (regardless of initial 25(OH)D status). Results were similar, albeit weaker, for females.

**Conclusions:**

Using two different methods to triangulate evidence, our findings suggest moderate to small causal links between circulating 25(OH)D and grip strength.

Low vitamin D status is a widespread phenomenon (1) although status can be improved with supplementation. Muscle weakness, often indicated by low grip strength, is another public health concern. Its prevalence increases at older ages, and it is a key component of age-related conditions such as sarcopenia and frailty (2,3). The burden of muscle weakness is experienced by individuals, their families, and society, and due to the aging population, this global burden is expected to rise.

Vitamin D receptors have been identified in muscle cells (4,5), lending plausibility to the hypothesis that vitamin D plays a role in normal muscle function. While underlying mechanisms are not fully understood, current evidence suggests important genomic and non-genomic effects of vitamin D, with deficiency proposed to lead to increased muscle protein breakdown, impaired mitochondrial function, and greater muscle adiposity (6). Cellular and animal studies suggest that muscle responds to vitamin D (7), with the implication that supplementation could prevent/treat muscle weakness. However, the relationship between vitamin D status and muscle strength in humans is equivocal. For example, while a systematic review and meta-analysis of 29 randomized controlled trials (mean age: 61.1 years) reported a small benefit of vitamin D supplementation on muscle strength (8), a reanalysis removing data from two studies that have been retracted or had unresolved data irregularities found no relationship (9). Similarly, no evidence of effect was found in a recent trial of vitamin D supplementation over a 4-month period, among 40–80-year-old community-dwelling adults with low vitamin D status at baseline (10). There are several potential reasons for the lack of consensus regarding the relationship between vitamin D and strength including the fact that effects of supplementation may only become apparent after a long duration, with trials not of sufficient length. Observational studies of circulating 25-hydroxyvitamin D concentration (25(OH)D; an established marker for vitamin D status) and muscle strength may be limited by issues such as residual and unmeasured confounding by, for example, body composition. Thus, a recent Mendelian randomization (MR) analysis in UK Biobank was important in providing evidence of a modest association between higher lifetime 25(OH)D concentrations and stronger grip (11). While this MR study provided evidence of an association that was less subject to confounding, it assumed a linear dose–response relationship. However, observational studies suggest that the relationship could vary by age and underlying 25(OH)D exposure (8). Thus, examining associations assuming (i) stability across the life course and (ii) linearity, might not provide an accurate picture of the dose–response relationship between vitamin D and strength. It is therefore imperative to understand whether variation in vitamin D exposure causally influences muscle strength taking account of these two factors.

Given current knowledge gaps, we aimed to establish the causal role of vitamin D on grip strength using UK Biobank data. Our approach is one of triangulation (12), where we obtain more robust evidence by integrating results using different methodologies (observational and genetic). Each methodology has different key sources of potential bias that are unrelated to each other, and if the results from our different methodological approaches are consistent, it strengthens confidence with respect to the causality of our findings. In our observational analysis, we examined cross-sectional associations between circulating concentrations of 25(OH)D and grip strength. Acknowledging the suggestion that the effect of vitamin D supplementation on muscle strength is greater in persons with low baseline 25(OH)D (8), we allowed for nonlinear relationships in our analysis. To explore whether associations between 25(OH)D and strength vary by age (8), we reran analyses stratified by 4 age groups. To triangulate evidence, we used MR to estimate the strength of the association using genetic instruments for 25(OH)D; crucially, we used MR methods that allowed us to assess nonlinear effects of vitamin D on grip strength.

## Method

UK Biobank (13,14) is a prospective study of over 500,000 UK adults aged 37–73 years at recruitment (2006–2010). Participants recruited across the United Kingdom from National Health Service central registers, provided informed consent; ethical approval was given by the Northwest Multicentre Research Ethics Committee. The genome-wide association study (GWAS) used to identify our genetic instrument (described below) was primarily based on samples of European ancestry. Hence, we examine data from participants of European ethnicity and our analytical sample with valid genotype data, measures of grip strength, 25(OH)D, and no missing covariates was 368,890.

*Vitamin D* 25(OH)D concentrations were measured in blood samples collected at the initial assessment visit (2006–2010). The DiaSorin Liaison XL, a chemiluminescent immunoassay, was used for the quantitative determination of total 25(OH)D, with a validated range for the assay of 10 nmol/L to 375 nmol/L. The average within-laboratory coefficient of variation (and standard deviation [*SD*]) ranged from 5.04 (4.73) to 6.14 (2.21) (15).

*Grip strength* in kilograms was assessed, once in each hand while seated, at the initial visit, using a Jamar J00105 hydraulic hand dynamometer (16). We examined the maximum recorded value (greater than 0).

*Covariates* were identified a priori and included established determinants of circulating 25(OH)D and potential confounders. Calendar month of assessment visit and vitamin D supplementation (consumed a multivitamin or vitamin D supplement in the previous 24 hours; regular supplementation with vitamin D or self-reported medical treatment with vitamin D products) were considered established determinants of 25(OH)D; potential confounders included age, smoking status, body mass index (BMI; (kg/m^2^) ascertained following standardized protocols (13)) and the Townsend score (area level indicator of socioeconomic deprivation) (17).

*Genetic instrument for circulating 25(OH)D concentration* was constructed using 6 variants that attained genome-wide significance (*p* < 5 × 10^−8^) for circulating levels of 25(OH)D in a GWAS of 79,366 European-ancestry individuals (18). We used linkage disequilibrium clumping in PLINK 1.9 to ensure these variants were independent (*r*^2^ ≤ 0.01, 250 kb). In the GWAS, these variants explained 2.84% of the variability in circulating 25(OH)D; see Supplementary Table 1 for further information. Our genetic instrument is a weighted genetic score constructed by computing the weighted average of the number of 25(OH)D increasing alleles for an individual, where the weight for each single nucleotide polymorphism (SNP) was the effect estimate of the association of the SNP with circulating 25(OH)D (on the natural log scale) reported in the GWAS (18).

### Statistical Methods

Due to marked sex differences in the distribution of grip strength (19), we chose a priori to sex-stratify all analyses.

#### Observational

The associations between circulating 25(OH)D concentration and grip strength were examined by fitting linear regression models, adjusting for the covariates listed above. Fractional polynomials were used to determine the appropriate functional form for circulating 25(OH)D concentrations (20). Using likelihood ratio tests, the best-fitting fractional polynomial was determined by comparing the best-fitting fractional polynomials of 1 and 2 degrees. We compared model fit between the best-fitting fractional polynomial and linear models, taking *p* < .05 to indicate a nonlinear association. To assess whether associations between 25(OH)D and grip strength vary by age, we reran analyses stratified by age groups representing approximate fourths of the population (ie, <50 years, 50–59 years, 60–64 years, 65 years+), with tests of interaction between age and circulating 25(OH)D concentration formally assessed.

#### Nonlinear MR

We used the fractional polynomial method to capture nonlinear genetic associations (21), with a reference point at 50 nmol/L (22). We stratified our sample into 100 strata using the doubly ranked method, whereby we first ranked participants into pre-strata according to their level of the weighted genetic score and then ranked participants within each pre-stratum according to their level of 25(OH)D (23). Within each stratum, we estimate the localized average causal effect (LACE), which is the ratio of coefficients of the regressions of the weighted genetic score on grip strength within the stratum to the regression of the weighted genetic score on  25(OH)D in the whole population. Both regressions were adjusted for circulating 25(OH)D determinants (month of blood draw, vitamin D supplementation) and genetic ancestry (10 genetic principal components). Next, because the causal effect of circulating 25(OH)D on grip strength (ie, LACE) is an estimate of the slope of the exposure–outcome relationship, we regressed stratum-specific mean 25(OH)D  against LACE estimates, using a fractional polynomial function (selecting the best-fitting model based on the likelihood ratio test). The integral of the selected fractional polynomial represents the relationship between circulating 25(OH)D and grip strength. The fractional polynomial test is reported for non-linearity, which compares the best-fitting fractional polynomial of degree 1 against the linear model (21). When there was no evidence favoring a nonlinear relationship (over a linear relationship) between 25(OH)D and grip strength, we performed a linear MR using the weighted genetic score as our instrument.

### Sensitivity Analysis and Instrument Validation

Our MR analyses could be biased in the presence of horizontal pleiotropy, that is, where the genetic instrument influences grip strength through pathways other than via circulating 25(OH)D. To assess the potential for pleiotropy, we examined whether our genetic instrument was associated with potential confounders in the entire sample. We also reran our MR analyses using a larger weighted genetic score, based on a more recent 25(OH)D GWAS (24) which included UK Biobank participants (details given in Supplementary Text A).

## Results

Mean maximum grip strength was higher in males (41.9 kg; *SD* = 8.9 kg) than females (25.2 kg; *SD* = 6.3 kg), whereas mean 25(OH)D was approximately 50 nmol/L in both sexes (Table 1).

**Table 1.** Characteristics (mean [*SD*]/*N* [%]) of UK Biobank Study Participants Included in Analyses

	Males	Females
*N*	172,171 (46.7%)	196,719 (53.3%)
Outcome		
Grip strength (kg)*	41.9 (8.9)	25.2 (6.3)
Exposure		
25(OH)D (nmol/L)	49.9 (21.1)	49.8 (20.9)
Determinants of 25(OH)D		
Month of blood draw		
January–March	43,655 (25.4)	47,179 (24.0)
April–June	48,896 (28.4)	58,624 (29.8)
July–September	40,104 (23.3)	47,564 (24.2)
October–December	39,516 (23.0)	43,352 (22.0)
Vitamin D supplementation^†^		
No	164,311 (95.4)	179,023 (91.0)
Yes	7 860 (4.6)	17,696 (9.0)
Potential confounders		
Age (years)‡	57.7 (39–73)	57.0 (40–71)
BMI (kg/m²)	27.8 (4.23)	27.0 (5.15)
Townsend deprivation index^§^	−1.54 (2.98)	−1.59 (2.88)
Smoking		
Never	84,488 (49.1)	117,122 (59.5)
Previous	67,608 (39.3)	62,675 (31.9)
Current	20,075 (11.7)	16,922 (8.6)

*Notes*: BMI = body mass index.

*Maximum of left and right hand measures; ^†^defined as a positive response to (i) taking a multivitamin or vitamin D supplement in the last 24 hours, (ii) regular supplementation with vitamin D, or (iii) self-reported medical treatment with vitamin D products, eg, calciferol, ergocalciferol, calcifediol, calcitriol (oral or injection; with or without calcium); ‡Range; ^§^Positive values indicate areas with high deprivation; negative values indicate relative affluence.

### Observational Associations: Measured 25(OH)D–Grip Strength

**Figure 1.**
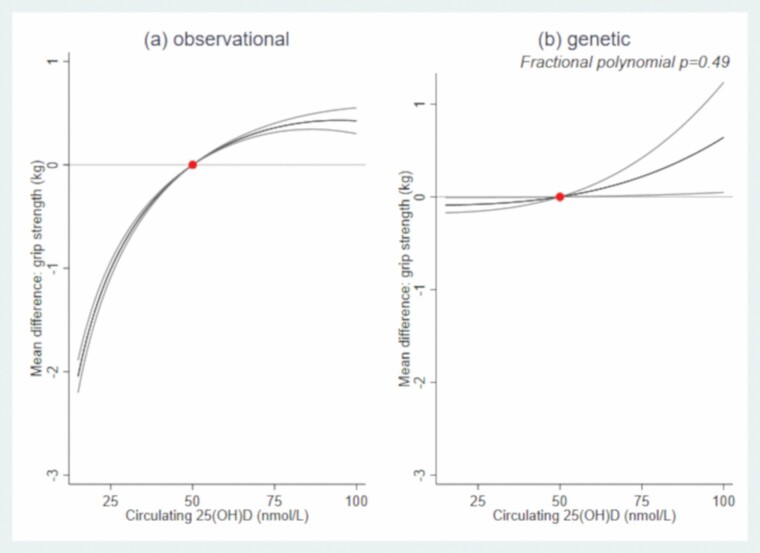
Mean differences (95% CI) in grip strength (kg) by circulating 25-hydroxyvitamin D: (A) observational and (B) genetic associations in males. The dot represents the reference point of circulating 25-hydroxyvitamin D (50 nmol/L). Observational associations adjusted for potential confounders (smoking, BMI, deprivation, and age) and established determinants of circulating 25(OH)D (vitamin D supplementation and month of blood draw). Genetic associations were adjusted for established determinants of circulating 25(OH)D (vitamin D supplementation and month of blood draw) and genetic ancestry (ie, 10 genetic principal components). BMI = body mass index; CI = confidence interval.

**Figure 2.**
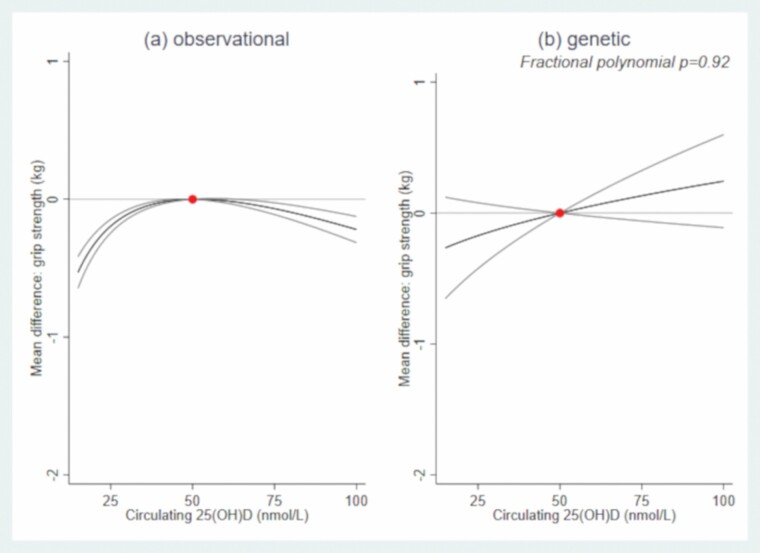
Mean differences (95% CI) in grip strength (kg) by circulating 25-hydroxyvitamin D: (A) observational and (B) genetic associations in females. The dot represents the reference point of circulating 25-hydroxyvitamin D (50 nmol/L). Observational associations adjusted for potential confounders (smoking, BMI, deprivation, and age) and established determinants of circulating 25(OH)D (vitamin D supplementation and month of blood draw). Genetic associations were adjusted for established determinants of circulating 25(OH)D (vitamin D supplementation and month of blood draw) and genetic ancestry (ie, 10 genetic principal components). BMI = body mass index; CI = confidence interval.

**Figure 3.**
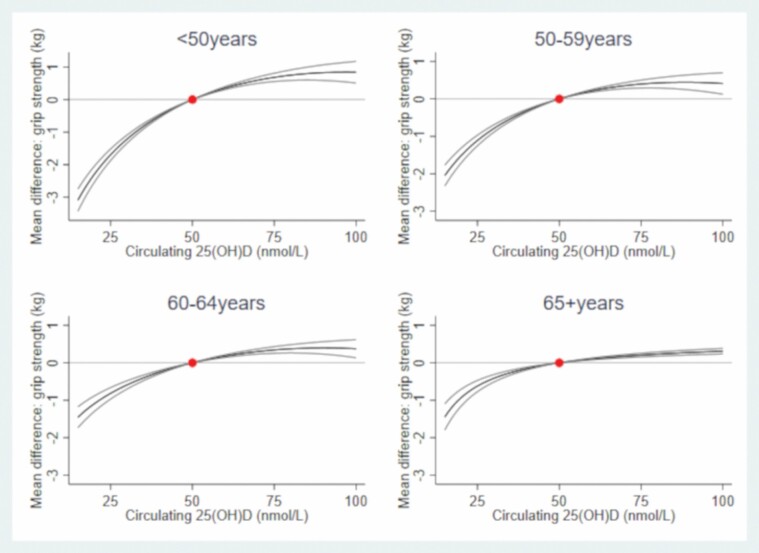
Mean differences (95% CI) in grip strength (kg) by circulating 25-hydroxyvitamin D from age-stratified observational analysis in males. The dot represents the reference point of circulating 25-hydroxyvitamin D (50 nmol/L); associations adjusted for potential confounders (smoking, BMI, deprivation, and age) and established determinants of circulating 25(OH)D (vitamin D supplementation and month of blood draw). BMI = body mass index; CI = confidence interval.

**Figure 4.**
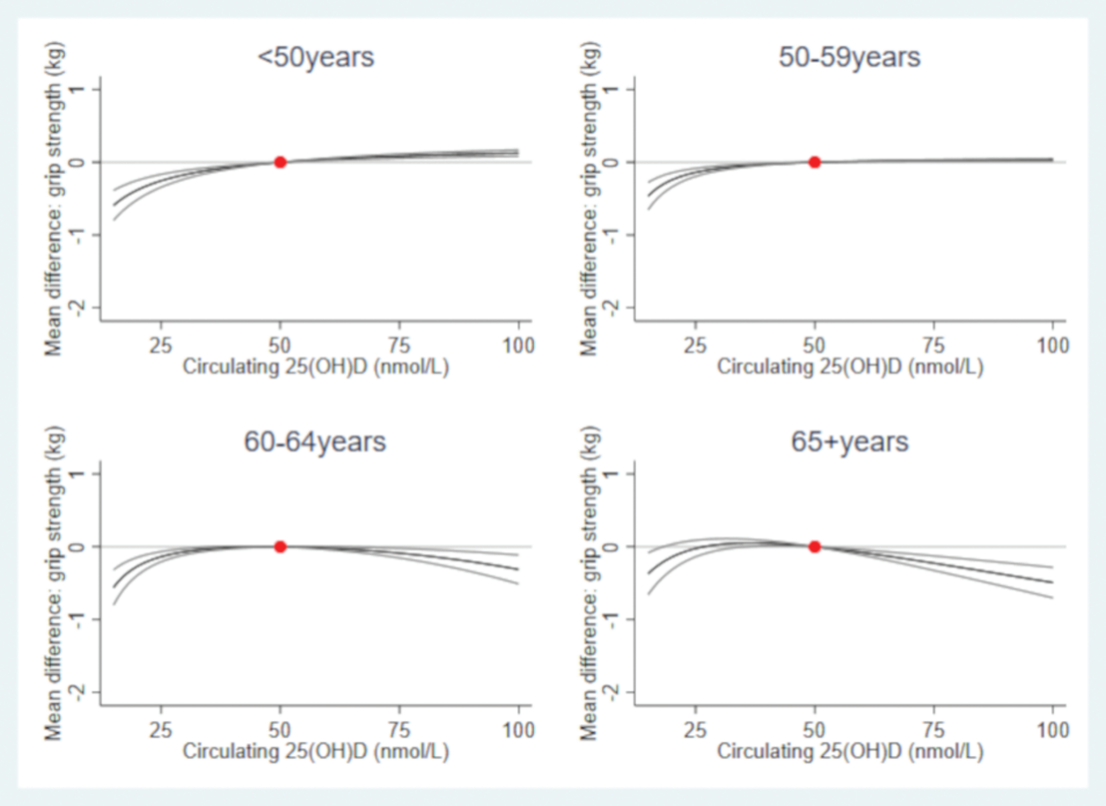
Mean differences (95% CI) in grip strength (kg) by circulating 25-hydroxyvitamin D from age-stratified observational analysis in females. The dot represents the reference point of circulating 25-hydroxyvitamin D (50 nmol/L); associations adjusted for potential confounders (smoking, BMI, deprivation, and age) and established determinants of circulating 25(OH)D (vitamin D supplementation and month of blood draw). BMI = body mass index; CI = confidence interval.

In both sexes, there was evidence of a nonlinear association (*p*_nonlinear_ < .001) between 25(OH)D and grip strength (Figures 1A and 2A; Supplementary Table 2). For example, compared to males with 50 nmol/L of circulating 25(OH)D, males with circulating 25(OH)D  at 25 nmol/L had 1.01 kg (95% confidence interval [CI]: 0.93, 1.08) weaker grip, those with circulating 25(OH)D at 75 nmol/L had 0.36 kg (0.31, 0.40) stronger grip. Compared to females with 50 nmol/L, females with circulating 25(OH)D at 25 nmol/L had 0.19 kg (0.15, 0.24) weaker grip; females with 25(OH)D at 75 nmol/L also had weaker grip (by 0.07 kg (0.03, 0.11)). In both sexes, there was evidence that circulating 25(OH)D–grip strength associations varied by age (*p*_interaction_ < .001; Supplementary Table 3), whereby the circulating 25(OH)D–grip strength slope was shallower (i.e., less steep) with increasing age (Figures 3 and 4).

### Linear and Nonlinear MR Associations: Genetically Predicted 25(OH)D–Grip Strength

In males and females, the linear and nonlinear models fitted the data similarly well (Figures 1B and 2B, *p*_nonlinear_ = .49(males)/.92(females)).  Based on the linear MR analysis, 25 nmol/L higher circulating 25(OH)D was associated with 0.25 kg (−0.05, 0.55) and 0.14 kg (−0.07, 0.36) greater grip in males and females, respectively (regardless of initial 25(OH)D status; Supplementary Table 4).

### Sensitivity Analysis and Instrument Validation

In our analytical sample, our weighted genetic score explained 2.61% of the variability in circulating levels of 25(OH)D in males and 2.37% in females. The *F*-statistic was 4 615.22 (males) and 4 772.91 (females). There was some evidence that our weighted genetic score was associated with potential confounders in UK Biobank, albeit differently by sex (Supplementary Table 5). When we reran the nonlinear and linear MR using the larger instrument, results were broadly similar to those reported above (Supplementary Text A). For example, the linear and nonlinear models fitted the data similarly well (*p*_non-linear_ = .30(males)/.11(females); Supplementary Figure 1), and a 25 nmol/L higher circulating 25(OH)D was associated with 0.28 kg (0.04, 0.52) and 0.03 kg (−0.14, 0.20) greater grip in males and females respectively (Supplementary Table 4).

## Discussion

We investigated associations between circulating 25(OH)D and grip strength in UK Biobank using two complementary approaches and examined whether non-linear associations were evident. While our observational and genetic analyses did not concur with respect to the shape of the 25(OH)D–grip strength association, they agreed in terms of noting a stronger association in males compared to females and generally a positive (ie, increasing) relationship between 25(OH)D and grip strength in males. As such, using two different methods to triangulate evidence, our findings suggest causal links between circulating 25(OH)D and grip strength, but, in terms of magnitude, our findings suggest vitamin D has a moderate to small impact on grip strength.

Our study has several strengths. For example, our MR associations are subject to less confounding and reverse causation inherent in cross-sectional observational analysis and, UK Biobank’s large sample size allowed us to increase the statistical power of our analysis, mitigating against weak instrument bias (25,26). Our focused 25(OH)D genetic instrument, based on 6 SNPs, provided strong statistical power, instrument strength, and biological specificity, minimizing the potential for bias due to the use of weak instruments and horizontal pleiotropy arising from using many variants that do not have specific effects on vitamin D pathways. There was only weak evidence that our focused score was associated with potential confounders, providing empirical evidence against horizontal pleiotropy although unmeasured confounding may exist. Moreover, when we repeated our genetic analysis with a larger instrument based on 98 SNPs, our results were near identical, providing further evidence of the robustness of our MR findings. Our observational analysis was cross-sectional, and although direction of causality cannot be inferred from such study designs, links between grip strength and subsequent 25(OH)D concentrations in this relatively young and healthy population are less likely to be operating than associations in the direction we have assumed. However, our findings do not negate the potential for a causal association from muscle weakness to low levels of 25(OH)D because, for example, low muscle strength may be associated with spending less time outdoors. Our observational analysis could also be affected by residual confounding, for example, while we accounted for BMI, we did not consider lean and fat mass (which could confound the 25(OH)D–strength association (27,28)). Our 25(OH)D analyses are limited in their ability to elucidate causal effects of the biologically active form of vitamin D, 1,25-dihydroxyvitamin D (1,25-OH2D), which correlates better with muscle strength than 25(OH)D (29). However, studies suggest the association between 25(OH)D and 1,25-OH2D with sarcopenia are independent (30), thus, their influence on muscle strength may also be independent. We were limited to using grip strength as our single measure of muscle strength as the strength of other muscles has not been assessed in UK Biobank. Although grip strength is a convenient and commonly used proxy for overall body strength and the Jamar dynamometer has good reliability and reproducibility (31), we acknowledge that grip strength is a measure of upper limb strength and evidence on whether it is an adequate proxy for overall muscle strength is equivocal (32,33). In addition, due to limited data availability, it was not feasible to examine other aspects of muscle, such as muscle mass. Our analysis was restricted to white Europeans, and we are unable to extrapolate to other ethnic groups, which is particularly relevant because markers of vitamin D metabolism vary significantly by ethnicity (34). Additionally, patterns of selection into studies such as UK Biobank (35) can induce collider bias. Even modest influences on selection could lead to biased estimates (36), and we acknowledge that such biases can affect both observational and MR analyses (37). Therefore, future work needs to assess whether our findings replicate in other samples including those representing other ethnic groups and, potentially, geographical regions.

To our knowledge, this is the first study triangulating evidence on the shape of the 25(OH)D–grip strength association, and while our observational and genetic analyses did not concur with respect to the shape, the analyses did concur with respect to suggesting vitamin D has a moderate to small impact on grip strength. For example, compared to males with 50 nmol/L circulating 25(OH)D, males with 75 nmol/L had 0.36/0.25 kg stronger grip; males with 25 nmol/L had 1.01/0.25 kg weaker grip (observational/genetic association). While severe vitamin D deficiency is rare, low status is common: 24%–40% of American, Canadian and European populations have circulating 25(OH)D levels below 50 nmol/L (38); and 13%–16% of UK adults have 25(OH)D levels below 25 nmol/L (39). Therefore, at the population level, our findings have important implications.

Our cross-sectional observational results conflict with previous reports that the relationship between vitamin D supplementation and muscle strength is stronger at older ages (8). However, our observational analysis was limited in terms of the age range examined and our MR analysis cannot inform on whether the shape and strength of the relationship between circulating 25(OH)D and grip varies by life stage. Thus, future work needs to examine if the magnitude and shape of the 25(OH)D–strength relationship varies over a wider age range. It is important to also appreciate that our observational and genetic findings are not interchangeable: the former examines 25(OH)D at the time of blood draw, whereas the latter represents lifelong differences in 25(OH)D levels, rather than a more transient short-term exposure; this difference may explain (in part) why our observational and genetic findings do not concur with respect to the shape of the 25(OH)D–strength relationship. Finally, our findings concur with a previous MR demonstrating a beneficial effect of 25(OH)D on grip strength in UK Biobank (11); however, we add new knowledge by considering the shape of the causal relationship between 25(OH)D concentrations and grip strength and quantifying the scale of the association.

Our findings have potential implications for the etiological understanding of muscle weakness and its treatment and prevention. Vitamin D supplementation is of considerable interest, as demonstrated by recent calls for evidence on how to improve the vitamin D status of the English population (39). While our results suggest causal links between circulating 25(OH)D and grip strength, they imply that vitamin D supplementation may only have a moderate to small effect on strength.

## Supplementary Material

glac255_suppl_Supplementary_Material
