# Supplementary material for: Linear and Nonlinear Associations Between Vitamin D and Grip Strength: A Mendelian Randomization Study in UK Biobank
Source: J Gerontol A Biol Sci Med Sci. 2022 Dec 25;78(8):1483–8. doi: 10.1093/gerona/glac255 (PMC10395562; doi:10.1093/gerona/glac255)
Supplement: glac255_suppl_Supplementary_Material [file glac255_suppl_supplementary_material.pdf]

Supplementary text A

We reran our MR analyses using a larger weighted genetic score, based on a more recent 25(OH)D GWAS[1] which included UK Biobank participants. After performing linkage disequilibrium clumping in PLINK1.9 to ensure that included SNPs were independent (r2≤0.01, 250kb, reference haplotype data originated from the publicly released Phase 3 data from the 1000 Genomes Project[2]), our weighted genetic risk score compromised 98 SNPs (see Supplementary Table 6 for details). In our analytical sample, this weighted genetic score explained 4.16% of the variability in circulating levels of 25(OH)D in males and 3.85% in females. The F-statistic was 7,481.78 (males) and 7,878.08 (females). For MR analysis results (non-linear and linear) see Supplementary Table 4 and Supplementary Figure 1.

Supplementary Table 1: Summary statistics describing SNP associations with 25(OH)D in original GWAS*

				Associations with:	ln(25(OH)D) in original GWAS*	
SNP	Chr	Nearest Gene	Main allele	Other allele	Beta	SE
rs3755967	4	GC	C	T	0.0892	0.0023
rs12785878	11	DHCR7	T	G	0.0363	0.0022
rs10741657	11	CYP2R1	A	G	0.0308	0.0022
rs10745742	12	AMDHD1	T	C	0.0165	0.0022
rs8018720	14	SEC23A	G	C	0.0168	0.0029
rs17216707	20	CYP24A1	T	C	0.0263	0.0027

SNP: single nucleotide polymorphism; SE: standard error; *Effect estimates from discovery GWAS (Jiang et al Nat Com (2018) 9:260) were from linear regressions of variants on natural-log transformed 25(OH)D. Used as weights in our analysis; see methods for details.

Supplementary Table 2: Model coefficients (95% CI), from fractional polynomials, for observational and genetic associations of circulating 25(OH)D with grip strength (kg)

	Males	Females
	Observational associations	
$25(OH)D^{-0.5}$	-17.90 (-19.32,-16.48)	
$25(OH)D^{3}$	$-3.61*10^{-7}$ ($-5.27*10^{-7}$,$-1.94*10^{-7}$)	
ln(25(OH)D)		1.72 (1.32,2.12)
$25(OH)D^{0.5}$		-0.48 (-0.61,-0.36)
	Genetic associations	
$25(OH)D^{3}$	$7.33*10^{-7}$ ($0.55*10^{-7}$,$14.12*10^{-7}$)	
$25(OH)D^{0.5}$		0.08 (-0.04,0.20)

Observational associations adjusted for determinants of circulating 25(OH)D (month of blood draw and Vitamin D supplementation) and potential confounders (age, BMI, deprivation, and smoking). Genetic associations were adjusted for determinants of circulating 25(OH)D (month of blood draw and Vitamin D supplementation) and genetic ancestry (i.e., 10 genetic principal components). Observational association parameters in the table were combined to provide illustrative examples in the Results. For example, difference in mean grip strength in females with 50nmol/L and 25nmol/L from observational analysis is: $(1.72*\ln(50) - 0.48*50^{0.5}) - (1.72*\ln(25) - 0.48*25^{0.5}) = 0.19$kg.

Supplementary Table 3: Model coefficients (95% CI), from fractional polynomials, for observational associations of circulating 25(OH)D with grip strength (kg), stratified by age*

Age	<50	50-59	60-64	65+	
		**Males**			$p_{interaction}$**
N (%)	36834 (21.4)	54165 (31.5)	43613 (25.3)	37559 (21.8)	<0.001
ln(25(OH)D)	2.85 (2.48,3.22)	1.93 (1.62,2.25)	1.27(1.01,1.52)		
$25(OH)D^2$	$-1.52*10^{-4}$ ($-2.17*10^{-4}$, $-8.59*10^{-5}$)	$-1.24*10^{-4}$($1.82*10^{-4,}$ $6.6*10^{-5}$)			
$25(OH)D^{-0.5}$					
$25(OH)D^3$			$-5.75*10^{-7}$ ($-9.37*10^{-7}$, $-2.19*10^{-7}$)		
$25(OH)D^{-1}$				-31.0 (-38.5,-23.5)	
		**Females**			$p_{interaction}$**
N (%)	45253 (23.0)	67004 (34.1)	48519 (24.7)	35943 (18.3)	<0.001
$25(OH)D^{-1}$	-12.69 (-17.09,-8.29)				
ln(25(OH)D)				3.87 (2.11,5.62)	
$25(OH)D^{0.5}$					
$25(OH)D^{-2}$		-115.0 (-161.5,-68.6)	-150.5 (-212.3,-88.7)		
$25(OH)D^3$			$-4.07*10^{-7}$ ($-6.40*10^{-7}$,$-1.74*10^{-7}$)		
$ln(25(OH)D)^2$				-0.54 (-0.77, -0.30)	

*Models adjusted for determinants of circulating 25(OH)D (month of blood draw and Vitamin D supplementation) and potential confounders (age (continuous variable), BMI, deprivation and smoking); **$p_{interaction}$ is the p-value for the interaction between 25(OH)D (as modelled in Supplementary Table 2, observational analysis) and age

Supplementary Table 4: Model coefficients (95% CI) for linear genetic associations of circulating 25(OH)D with grip strength (kg)*

	Males	p-value	Females	p-value
Main analysis: 6 SNP instrument	0.0099 (-0.0022,0.022)	0.11	0.0058 (-0.0028,0.014)	0.19
Supplementary analysis: 98 SNP instrument	0.011 (0.0017,0.02)	0.02	0.0013 (-0.0054,0.0079)	0.71

*Genetic associations were adjusted for determinants of circulating 25(OH)D (month of blood draw and Vitamin D supplementation) and genetic ancestry (i.e., 10 genetic principal components). Associations represent change in grip strength per nmol/L increase in 25(OH)D; coefficients were multiplied by 25 to provide examples in text regarding change in grip strength per 25nmol/L increase in 25(OH)D.

Supplementary Table 5: Mean (SD) of 25(OH)D weighted genetic score by potential confounders in UK Biobank

	Males		Females	
	N(%)	Mean (SD)	N(%)	Mean (SD)
Age (years)				
<50	36834 (21.4)	1.25 (0.31)	45253 (23.0)	1.25 (0.31)
50-59	54165 (31.5)	1.25 (0.31)	67004 (34.1)	1.25 (0.31)
60-64	43613 (25.3)	1.25 (0.31)	48519 (24.7)	1.25 (0.31)
65+	37559 (21.8)	1.25 (0.32)	35943 (18.3)	1.25 (0.31)
p-value*	0.07		0.54	
BMI (kg/m²)				
<25	43227 (25.1)	1.25 (0.31)	79220 (40.3)	1.25 (0.31)
25-30	85233 (49.5)	1.25 (0.31)	72045 (36.6)	1.25 (0.31)
≥30	43711 (25.4)	1.25 (0.31)	45454 (23.1)	1.25 (0.31)
p-value*	0.31		0.04	
Townsend deprivation index quartiles				
Q1 (least deprived)	34431 (20.0)	1.25 (0.31)	39303 (20.0)	1.25 (0.31)
Q2	34434 (20.)	1.25 (0.32)	39377 (20.0)	1.25 (0.31)
Q3	34432 (20.0)	1.25 (0.31)	39350 (20.0)	1.25 (0.32)
Q4	34436 (20.0)	1.25 (0.32)	39344 (20.0)	1.25 (0.31)
Q5 (most deprived)	34438 (20.0)	1.25 (0.31)	39345 (20.0)	1.25 (0.31)
p-value*	0.005		0.53	
Smoking				
Never	84488 (49.1)	1.25 (0.32)	117122 (59.5)	1.25 (0.31)
Previous	67608 (39.3)	1.25 (0.31)	62675 (31.9)	1.25 (0.31)
Current	20075 (11.7)	1.25 (0.31)	16922 (8.6)	1.24 (0.31)
p-value*	0.35		0.02	

*p-value from regression of each variable and weighted genetic score

Supplementary Table 6: Summary statistics describing SNP associations with 25(OH)D in larger GWAS*

SNP	Chr	Main allele	Other allele	Beta*	SE
rs12803256	11	G	A	0.104	0.002
rs28364331	4	G	A	0.063	0.007
rs2762943	20	G	T	0.045	0.004
rs11591147	1	T	G	0.045	0.007
rs964184	11	C	G	0.041	0.003
rs7604788	2	T	C	0.035	0.005
rs7412	19	T	C	0.033	0.004
rs2074735	22	C	G	0.028	0.004
rs142158911	19	A	G	0.027	0.003
rs72997623	11	A	C	0.026	0.004
rs10426	19	A	G	0.025	0.002
rs77532868	10	T	C	0.023	0.004
rs11076175	16	G	A	0.023	0.003
rs73413596	12	C	T	0.022	0.004
rs1260326	2	C	T	0.021	0.002
rs1149605	11	C	T	0.02	0.003
rs7528419	1	G	A	0.02	0.002

rs13284054	9	C	T	0.018	0.003
rs4364259	4	A	G	0.017	0.002
rs2037511	18	A	G	0.017	0.003
rs804281	8	G	A	0.016	0.002
rs2952289	17	T	C	0.016	0.002
rs10908465	1	T	C	0.016	0.002
rs3925446	10	A	G	0.015	0.002
rs6672758	1	T	C	0.015	0.002
rs28692966	8	A	G	0.014	0.002
rs9861009	3	C	T	0.014	0.002
rs7569755	2	A	G	0.014	0.002
rs7784802	7	T	A	0.013	0.002
rs8113404	19	T	C	0.012	0.002
rs61891388	11	G	T	0.012	0.002
rs1038165	12	T	C	0.011	0.002
rs4418728	10	T	G	0.011	0.002
rs11127186	2	C	T	0.011	0.002
rs9476310	6	T	C	0.011	0.002
rs11606	19	G	C	0.011	0.002
rs9490317	6	C	T	0.011	0.002
rs10070734	5	C	T	0.01	0.002
rs4738684	8	G	A	0.01	0.002
rs12881545	14	C	G	0.01	0.002
rs2725371	8	G	A	0.009	0.002
rs13104260	4	A	G	0.006	0.002
rs61883501	11	A	C	0.001	0.005
rs590215	18	C	T	0.005	0.002
rs76798800	1	G	T	0.009	0.002
rs11264322	1	G	A	0.01	0.002
rs7149014	14	T	C	0.011	0.002
rs727857	2	G	A	0.011	0.002
rs2710651	2	G	A	0.011	0.002
rs6966728	7	C	T	0.011	0.002
rs10908419	1	G	A	0.011	0.002
rs10887718	10	C	T	0.011	0.002
rs62007299	15	G	A	0.012	0.002
rs10454087	17	C	T	0.012	0.002
rs11182428	12	T	C	0.012	0.002
rs1047891	2	C	A	0.012	0.002
rs6003456	22	T	A	0.012	0.002
rs31612	5	T	C	0.012	0.003
rs4616820	4	C	T	0.012	0.002
rs7522116	1	C	T	0.013	0.002
rs325384	15	C	T	0.013	0.002
rs77924615	16	G	A	0.013	0.003
rs28374650	6	C	T	0.013	0.002
rs13060130	3	C	T	0.014	0.003
rs75741381	7	C	G	0.014	0.003
rs10085881	7	T	C	0.014	0.002
rs2346264	7	A	C	0.014	0.002
rs4575545	16	G	A	0.015	0.002

rs867772	1	A	G	0.015	0.002
rs11732896	4	G	A	0.015	0.002
rs3849374	2	G	C	0.016	0.003
rs4121823	18	T	A	0.017	0.003
rs78151190	6	A	C	0.018	0.003
rs6782190	3	G	A	0.019	0.002
rs55829990	15	T	C	0.019	0.002
rs12317268	12	A	G	0.019	0.003
rs78649910	4	T	A	0.02	0.003
rs2248551	6	G	A	0.02	0.003
rs35408430	1	C	T	0.021	0.002
rs12372115	12	G	T	0.021	0.004
rs2131925	1	G	T	0.021	0.002
rs2847500	11	G	A	0.022	0.003
rs12056768	8	T	G	0.022	0.002
rs4327060	16	C	T	0.023	0.004
rs11542462	16	G	A	0.024	0.003
rs2229742	21	G	C	0.024	0.003
rs8091117	18	C	A	0.024	0.004
rs72834856	6	T	G	0.025	0.004
rs261291	15	T	C	0.026	0.002
rs8018720	14	G	C	0.03	0.003
rs1800588	15	C	T	0.031	0.002
rs2207132	20	G	A	0.034	0.006
rs17216707	20	T	C	0.036	0.003
rs10859995	12	T	C	0.04	0.002
rs2012736	2	C	A	0.046	0.004
rs212100	19	T	C	0.06	0.003
rs1352846	4	A	G	0.184	0.002
rs116970203	11	G	A	0.344	0.006

SNP: single nucleotide polymorphism; SE: standard error; *Effect estimates from GWAS (Revez et al, Nat Commun (2020) 2;11(1)) were from linear regressions of variants on rank-based inverse-normal transformed 25(OH)D. Used as weights in our supplementary analysis; see methods for details.

Supplementary Figure 1: Mean differences (95% CI) in grip strength (kg) by circulating 25-hydroxyvitamin D in males and females (genetic associations using larger instrument, see Supplementary text A)

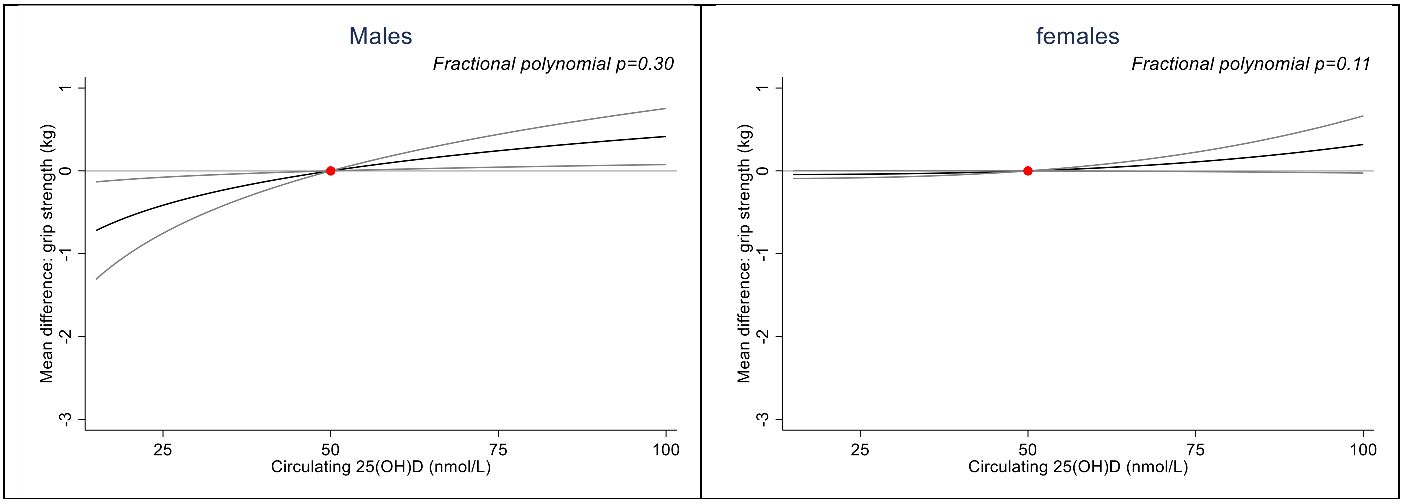

The dot represents the reference point of circulating 25-hydroxyvitamin D (50 nmol/L); associations were adjusted for established determinants of circulating 25(OH)D (vitamin D supplementation intake and month of blood draw) and genetic ancestry (i.e., 10 genetic principal components).

References in supplementary materials

[1] J. A. Revez *et al.*, "Genome-wide association study identifies 143 loci associated with 25 hydroxyvitamin D concentration," *Nat Commun,* vol. 11, no. 1, p. 1647, Apr 2 2020.

[2] C. Genomes Project *et al.*, "An integrated map of genetic variation from 1,092 human genomes," *Nature,* vol. 491, no. 7422, pp. 56-65, Nov 1 2012.
